# OVERCOMING THE CHALLENGES OF TECHNOLOGY IN GERONTOLOGICAL RESEARCH

**DOI:** 10.1093/geroni/igz038.827

**Published:** 2019-11-08

**Authors:** Jacob Lindsey, Elena Goodrich, Khoa Nguyen, Cierra Leon Guerrero, Colton Scavone, Supriya Pandya, Tanner Dorsey, Hiroko Dodge

**Affiliations:** Oregon Health & Science University, Portland, Oregon, United States

## Abstract

Many older adults express a lack of confidence in using technology and this can become a barrier to participating in technology heavy research. This presentation will introduce the face-to-face digital communication, electronic medication adherence tracking, and online recruitment technology used in the I-CONECT study (ClinicalTrials.gov: NCT02871921). In particular, we will demonstrate how the project has simplified enterprise video conferencing for in-home use, removed obstacles related to remote hardware troubleshooting, and further discuss how it has been received by older adults who have participated thus far. Finally we will cover particular hurdles related to I-CONECT (e.g., targeting social isolated older adults, aiming to recruit 50% of participants being African American older subjects living in the Detroit Metropolitan area). Our experience indicates that such high tech gerontological research is feasible given a creative and solution-focused research approaches and multi-disciplinary team.

